# The effect of reward and punishment on the extinction of attentional capture elicited by value-related stimuli

**DOI:** 10.1007/s00426-025-02115-2

**Published:** 2025-04-16

**Authors:** Francisco Garre-Frutos, Adriana Ariza, Felisa González

**Affiliations:** 1 Mind, Brain, and Behavior Research Center (CIMCYC), University of Granada, Granada, Spain; 2 Department of Experimental Psychology, University of Granada, Granada, Spain

## Abstract

Particular features of the stimuli that predict significant outcomes tend to capture our attention in a rather automatic and inflexible way. This form of attention has been described as a Pavlovian bias that mimics the phenomenon of sign-tracking described in animals, where reward-predictive cues become motivational magnets. In humans, Value-Modulated Attentional Capture (VMAC) refers to a phenomenon where distractors that signal high-value outcomes receive higher attentional priority. VMAC is particularly difficult to extinguish, showing a similar persistence often described in animal sign-tracking. In the present study, we evaluated to what extent VMAC would persist using a more specific extinction procedure than previous research, where instead of removing the possibility of obtaining rewards, the different discriminant stimuli that signal reward equate its value. Furthermore, we manipulated between experiments whether the high-value distractor predicted high-reward and high-punishment contingent to response accuracy (mimicking previous research; Experiment 1) or only high-reward (Experiment 2), and also explored the association of VMAC and its persistence with measures of emotional impulsivity employed in past research. Our results show that when both rewards and punishments are possible, VMAC does not extinguish after an extensive extinction stage, nor is it associated with measures of emotional impulsivity. When punishments were removed, we showed that VMAC gradually extinguished both in response times and accuracy and that the persistence of VMAC was significantly associated with positive urgency. We discussed these results on the potential of punishments to qualitatively alter learning and response strategies employed by participants.

**Supplementary Information:**

The online version contains supplementary material available at 10.1007/s00426-025-02115-2.

Multiple cues in the environment signal when a desired outcome is more likely to occur, providing information that potentially guides subsequent instrumental behavior and decision-making. In this sense, the cues possess predictive or informational value. For some individuals, or under certain conditions like experiencing a period of chronic stress or other negative affective states, these contextual cues may also acquire motivational value (e.g., drug-related stimuli associated with the rewarding effects of taking the drug) or incentive salience, acting as motivational magnets—the ‘wanting’ or motivational component of reward (Berridge, 2001; Berridge & Robinson, 1998, 2003). In this case, the cues may become desirable by themselves, influencing behavior in a way that exceeds its predictive or informational value, affecting behavior by its reward-related value instead, thus interfering with the individual’s goals. Interference with our current or long-term goals has been linked to the phenomenon of *sign-tracking*, originally proposed in the field of appetitive Pavlovian conditioning studies with animals as subjects (Hearst & Jenkins, 1974). For instance, a discrete and localizable stimulus (e.g., the insertion of a retractable lever) precedes the delivery of food pellets in the magazine, becoming a predictor of food. After several trials, some of the rats (goal-trackers; Boakes, 1977) show a conditioned response (magazine entry), looking for the reinforcer in the location where the food appears. For some other rats (sign-trackers), however, the stimulus additionally acquires incentive salience. As a result, the rats start to direct responses to the lever itself as if it were a subrogate of the food, such as sniffing and nibbling (Anselme et al., 2013) instead of looking for the food (the goal) in the magazine.

These two qualitatively different forms of behavior have typically considered two defined phenotypes: while goal-tracker animals tend to interact with a cue only conditional to a goal (i.e., the cue predicts the administration of an appetitive stimulus, such as food), sign-tracker animals persist in interacting with the cue even when the cue no longer predicts a relevant outcome. Individual differences in the tendency to show signs of goal-tracking conditioned responses have been explained through individual differences in how individuals translate the motivational attributes of the outcome to the cue (Flagel et al., 2007, 2011). Thus, sign-tracker animals persist in interacting with the cue because they learn to ‘like’ the cue (Berridge et al., 2009), which, after learning, has acquired motivational properties. As reviewed by Colaizzi et al. (2020), sign-tracking conditioned responses, unlike goal-tracking, exhibit greater resistance to extinction, susceptibility to reinstatement, and persistence, even if sign-tracking begins to produce an adverse outcome or loss in reward. Likewise, a goal-tracking response came to more rapidly match the changed contingencies in reversal learning compared to sign-tracking (see Iliescu et al., 2018).

In humans, sign-tracking has also been observed (Schad et al., 2020). Systematically pairing specific features of the stimuli with the prospect of reward elicits attentional capture of such features, which has been suggested as a form of attentional sign-tracking (Colaizzi et al. 2020; Le Pelley et al., 2015). For instance, in the seminal study by Anderson et al. (2011), participants first searched for a target defined by two possible colors among other non-distractors, one signaling high and the other low reward. In the second phase, participants were tasked to search for a singleton shape (i.e., a diamond between circles) and ignore other non-target stimuli (Theeuwes, 1992). Anderson et al. (2011) found that when the distractor associated with high reward in the previous stage was presented, the display response times (RTs) were higher than when the low-value distractor was presented.

The effect of feature-reward associations in the previous study has been explained by reinforcement of the attentional selection of the feature associated with reward (an Attentional Habit; Anderson, 2016; Failing & Theeuwes, 2018). Similar training-test paradigms have found that attentional biases toward high-value distractors occur even when the feature related to reward never requires a response (Bucker & Theeuwes, 2017, 2018; Mine & Saiki, 2015). These findings suggest that reward-related attentional biases rely merely on the feature-reward relationship and may be better explained by a Pavlovian attentional bias. One of the better demonstrations of the previous was provided by the study of Le Pelley et al. (2015). This study aimed to test whether reward-related attentional biases can be observed even if such a form of attention contradicts task goals. To that aim, they manipulated the magnitude of reward predicted by two different singleton distractors (high and low-value) while, as in Anderson et al. (2011), tasked participants to find diamond-shaped stimuli. Compared to Anderson et al. (2011), the associated feature was a response irrelevant distractor. Additionally, because the reward was contingent on overall performance in Le Pelley et al. (2015) (i.e., fast and accurate responses earned more points), being captured to the high-value distractor was counterproductive. In other words, attending to the high-value distractor would mean obtaining less reward than for the low-value stimuli. Even in such conditions, Le Pelley et al. (2015) found that RTs were slower when the high-value distractor was presented in the display, an effect they termed Value-Modulated Attentional Capture (VMAC).

Although the previous evidence highlights that VMAC can occur independently of task goals and stimulus-driven physical salience, in Le Pelley’s study, the associated feature also holds informational value, which has been proposed as a powerful driver of attention (Gottlieb et al., 2020). One alternative explanation is that informational value can trigger more strategic processes (i.e., attending to the distractor to obtain information about reward in a current trial). Thus, VMAC in Le Pelley et al. (2015) may not reflect a Pavlovian attentional bias. To test this idea, Watson et al., (2019a, b) replicated Le Pelley’s study but included a brief unrewarded test (two blocks of 24 trials) and showed that VMAC was observed even when the color feature was never task-relevant and no longer possesses informational value. In a subsequent study, we showed that even when you extend the unrewarded phase to match the length of the learning stage, VMAC persists throughout the whole unrewarded stage (Garre-Frutos et al., 2024).

The previous results indicated that VMAC may be explained as a Pavlovian bias that is especially difficult to extinguish (see also DeTommaso & Turatto, 2021; Le et al., 2025). Once learned, reward-related attentional biases are observed even a month after original learning episodes have taken place (Anderson & Yantis, 2013). Additionally, VMAC also has been shown to produce strong oculomotor capture effects (Failing et al., 2015; Theeuwes & Belopolsky, 2012) and is observed even when directly looking at the high-value distractor results in the omission of reward (Le Pelley et al., 2015). This oculomotor capture is persistent even though participants are explicitly instructed that looking at the high-value distractor will result in the omission of the reward (Pearson et al., 2015). Furthermore, VMAC is magnified when there are no reward omissions for looking at the reward (Pearson & Le Pelley, 2020), suggesting that participants avoid looking at the high-value distractor, but they only can reactively suppress attention to the high-value distractor (Pearson & Le Pelley, 2021). Le Pelley et al. (2015) were perhaps the first to propose that VMAC may be conceptualized as a measure of human sign-tracking. In this view, stimulus-reward pairings would cause the associated feature to gain motivational or incentive value (Berridge et al., 2009; Flagel & Robinson, 2017; Flagel et al., 2011), and just as in animal studies, features associated with reward would also become a motivational magnet at the attentional level. For instance, VMAC is resistant to the omission of reward, and sign-tracking also seems to be particularly unaffected by Pavlovian extinction procedures (Colaizzi et al., 2020; Fitzpatrick et al., 2019).

In the present work, we aim to extend previous results on the Pavlovian extinction of VMAC. The failure to observe an effect akin to extinction in this task might be due to the paradigm used: the complete omission of points (reward) in the second phase affected not only the high-value stimulus but the rest of them, completely changing the goal (to earn points) and context of the task. That might be a rather insensitive procedure; the persistence of the VMAC effect during the unrewarded phase could result from a task’s failure to engage the extinction process effectively; feedback on performance might have sustained behavior during the unrewarded phase. In the present studies (Experiments 1 and 2), we aimed to enhance the likelihood of observing the extinction effect by specifically extinguishing the increase in reward magnitude signaled by the high-value singleton without affecting the goal of the task nor the cue-reward contingency of the other stimuli^1^. Thus, in the second phase of the VMAC task, participants kept gaining points, but the prior high-value singleton did not signal extra points, acting as the rest of the distractors.

Besides modifying the extinction procedure, we also manipulated between experiments the use of punishment for incorrect responses. Thus, in Experiment 1, incorrect responses led to the loss of points that could have been otherwise earned (as in Garre-Frutos et al., 2024; Watson et al., 2019a, b). In the case of Experiment 2, we use the modified reward-only variant in which errors do not result in losses, only in the absence of points. This allows us to specifically study the effect of the high-value singleton reward-history experience on performance without confounding factors such as loss-related sensitivity processes or the effect of discriminative reinforcement, which are not central to the phenomenon of sign-tracking (see also, e.g., Albertella et al., 2019, 2020a, b).

Considering the above, one of our predictions was that the specific extinction procedure used in our Experiments 1 and 2—in which the increase in reward/punishment magnitude was extinguished—would make it more likely to observe a progressive decrease in the VMAC effect during the second phase of the task. In addition, we expected to find differences in the VMAC effect in acquisition (and perhaps in extinction) between Experiment 1 (with punishment) and Experiment 2 (reward-only variant), shedding light on the question of whether the observed attentional effects obey to reward learning specifically or to value learning more generally (Watson et al., 2019a, b). However, we were unable to make specific predictions regarding the effect of punishment-omission and thus this may be considered a more exploratory objective of our research.

Additionally, individual differences in the predisposition to exhibit sign-tracking behaviors have been theoretically linked to vulnerability to addiction (Flagel et al, 2009; Robinson & Berridge, 2008). A growing body of research in humans suggests substantial variability in the extent to which individuals attribute incentive salience to reward-related cues. Such variability underscores the interaction between repeated exposure to rewarding stimuli (e.g., drugs) and a pre-existing disposition toward attributing incentive salience to associated cues (Colazzi et al.,2020; Saunders & Robinson, 2013). In the same vein, there is considerable evidence suggesting that the VMAC effect can be conceptualized as a continuum measure of individual differences related to cognitive control and personality traits (Anderson, 2021; Le Pelley et al., 2024). Notably, susceptibility to reward-related cues varies significantly among individuals, and this variability has been related to personality traits such as impulsivity (Albertella et al., 2019, 2020b; see also Liu et al., 2021), as well as clinical conditions including substance use (Albertella et al., 2017, 2021; Anderson et al., 2013; Liu et al., 2021) or ADHD (Sali et al., 2018). In this context, the VMAC effect provides a tool for studying individual differences in the attribution of incentive salience to reward cues, with a particular focus on the persistence of reward-related attentional bias (Albertella et al., 2019).

Given the above, we were particularly interested in testing whether persistence of previously learned Pavlovian attentional biases would correlate with measures of emotional impulsivity, as measured by the S-UPPS-P questionnaire (Cyders et al., 2014). For instance, previous studies have failed to find significant associations between VMAC and overall impulsivity (as measured by the total S-UPPS-P score; Liu et al., 2021). However, Albertella et al. (2020b) found that two affect-driven impulsivity factors—the tendency to act rashly when experiencing intense negative (negative urgency, NU) or positive (positive urgency, PU) emotions–significantly correlated with the persistence of VMAC to the reverse of previously learned associations. In a similar vein, using another relatively pure assay of incentive salience (Pavlovian-to-instrumental transfer, PIT; Peciña & Berridge, 2013) NU has been reported to be negatively related to PIT devaluation (Hinojosa-Aguayo & González, 2020), suggesting that affective-impulsivity makes incentive salience more persistent and inflexible. Thus, in the present study we aimed to explore the association between both measures of emotional impulsivity with the acquisition of reward-related attentional biases and its persistence following extinction. Following previous research, we hypothesize that greater persistence of the VMAC effect during the extinction stage of the task will be associated with higher levels of emotional impulsivity.

## Experiment 1

In Experiment 1, the acquisition phase followed the procedure used by Watson et al., (2019a) and Garre-Frutos et al. (2024), where punishment followed incorrect responses, but we modified the extinction phase. Instead of eliminating the possibility of gaining points, we specifically extinguished the reward/punishment magnitude increase indicated by the high-value singleton. In this case, the high-value singleton now functioned as the low-value one, and participants continued to gain or lose points during this phase.

## Methods

### Participants

Based on a power analysis reported in the supplementary material (Figure S1), we aimed to recruit at least 100 participants. To recruit participants, our experiments were published on SONA, the official experiment platform of the University of Granada. Volunteers with normal or corrected-to-normal vision and Spanish as their mother tongue participated in the study. In this experiment, 103 participants completed the study (82 females, 20 males, and one non-binary), aged 18–29 (*M* = 20.5, *SD* = 2.3). Undergraduate students received course credits for their participation, and the top ten performers in each experiment, based on task performance, received €5 as additional compensation. The protocols of the experiments reported here were approved by the Ethics Committee on Human Research of the University of Granada (approval number 3022/CEIH/2022). The present experiment was not formally preregistered.

### Materials

The entire study was computerized. Three desktop computers were used, each equipped with a 21.5″ HD monitor with a resolution of 1920 × 1080 pixels. The following materials were used.

#### Short Spanish version of the UPPS-P impulsive behaviour scale (Cándido et al., 2012)

The scale comprises 20 4-point Likert-type items (1 = *strongly agree*, 4 = *strongly disagree*). As explained above, we were particularly interested in correlating the Negative and Positive Urgency subscales—whose reliability indices were Cronbach’s α = 0.81 and α = 0.59, respectively—, with VMAC. Pearson’s *r* correlations between VMAC and the rest of the scales, as well as the total score and a general urgency score (see Billieux et al., 2021; Riley & Smith, 2017) are presented in supplementary material (Table S7).

#### Value-modulated attentional capture (VMAC) task

**Fig. 1:**
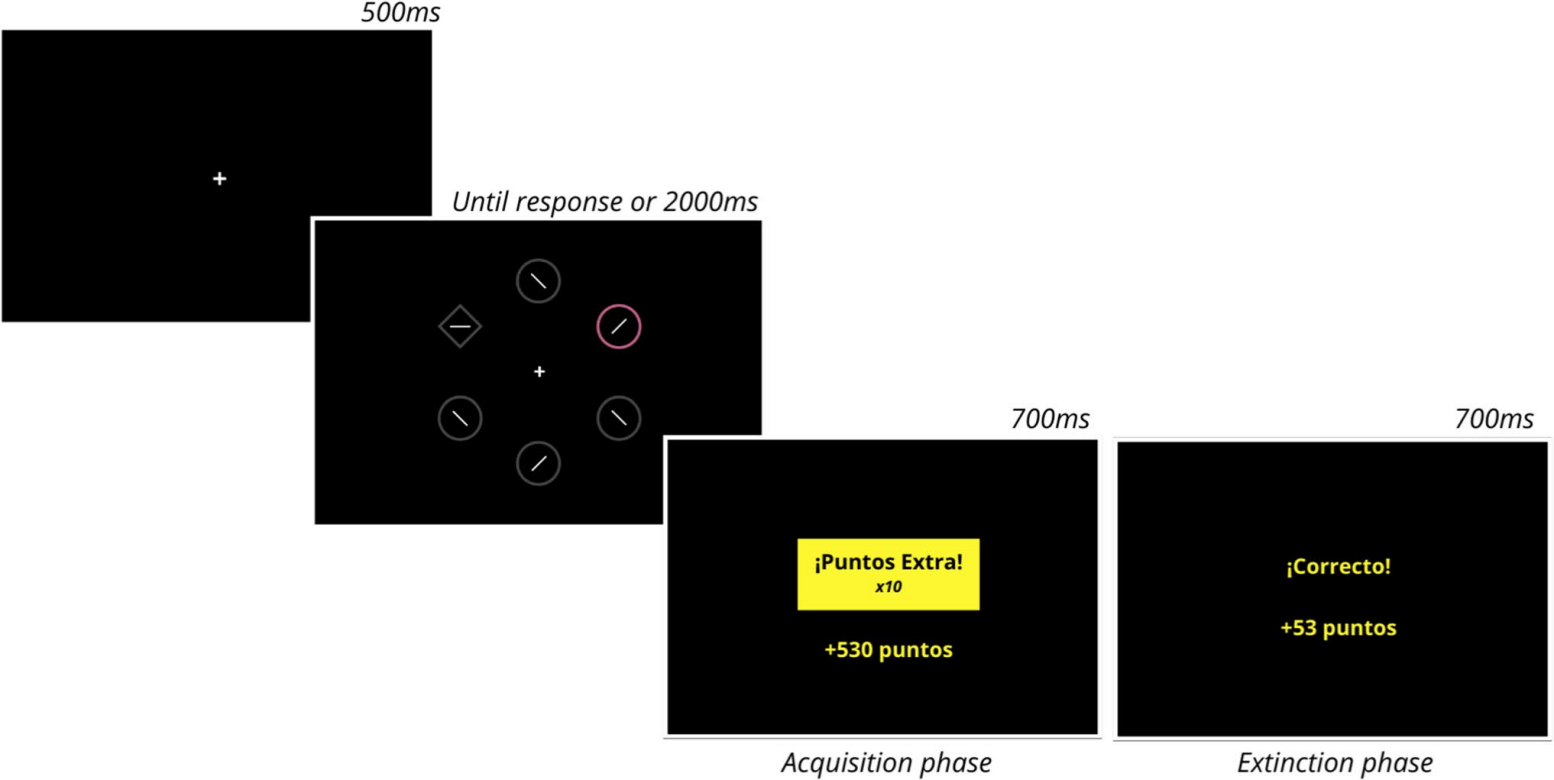
Graphical representation of the experimental procedure. The figure depicts an example of a High-value trial. In the first phase (the acquisition phase), the presence of the High-value singleton signaled a bonus trial, where points gained (and lost in Experiment 1) were multiplied by ten. Participants received specific feedback after each response. In the second phase (extinction), the High-value singleton no longer signaled an increased reward (or punishment); it became equivalent to the other singletons in the task

The task was programmed in JavaScript Psych and hosted in JATOS. It comprised two phases: acquisition, where two different value levels (High and Low) were available, and extinction, where the High-value condition was modified to equate to the Low-value condition. Each phase consisted of 12 blocks of 24 trials: 10 High-value trials, 10 Low-value trials, and 4 Absent trials (no cue signaled the magnitude of the reward, but it was still available). Each trial began with a fixation cross at the center of the screen, followed by a search display containing six shapes (2.3° × 2.3° visual angle) arranged in a circular layout. Five of the shapes were circles, each containing a segment tilted randomly 45° to the left or right. The sixth shape was a diamond (target), with its segment randomly oriented either horizontally or vertically. In most trials, one circle was colored (Singleton), while the other stimuli appeared gray on a black background. Two color pairs were used for High and Low Singletons (blue and orange, pink and green), randomly assigned across participants and Singletons. The shape location varied randomly in each trial. Trials ended after 2000 ms or once a response was emitted, with an inter-trial interval of 1200 ms (see Fig. 1).

Participants were instructed to respond as quickly and accurately as possible, indicating whether the segment inside the diamond was horizontal or vertical by pressing the C or G keys, respectively. Correct responses earned points, while incorrect responses resulted in point losses. The faster the response, the greater the amount of points was earned or lost. Specifically, during the acquisition phase, participants earned/lost 0.1 points for every millisecond their RT was below 1000 ms, except in High-value trials, where points were multiplied by 10. In the extinction phase, the High-value cue was extinguished: participants were instructed that the High-value cue now functioned as the Low-value cue. Feedback was displayed for 700 ms after each response (*Correct!: *+*[number of points earned] / Error: −[number of points lost]*).

### Procedure

Participants received a detailed description of the study and provided written informed consent to participate. Afterward, sociodemographic data were collected, and the questionnaire was completed. For the VMAC task, participants first completed a practice block of 24 trials, in which all stimuli were gray (Absent trials). Then, they received verbal and written instructions for the acquisition phase and completed 12 blocks of 24 trials each. Finally, they were instructed about the extinction phase as follows: “From now on, the [*High color*] circle no longer indicates the availability of extra points. You will gain or lose the points based on your speed, as in the case of trials where there are no colors or where the [*Low color*] circle appears.” They completed 12 additional blocks of 24 trials for the extinction phase. The entire experimental session lasted approximately 60 min, consisting of 15 min for questionnaires and 45 min for the VMAC task (5 min for instructions and practice, and 20 min per phase).

### Data analysis

Data were analyzed using the same approach as in Garre-Frutos et al. (2024). The acquisition and extinction phases were analyzed separately. We assessed the attentional capture effect (AC; RTs on low-valued singleton trials—RTs on absent singleton trials), the VMAC effect (RTs on high-valued singleton trials—RTs on low-valued singleton trials), and the development of both effects across blocks of trials.

RTs were analyzed using linear mixed models (LMMs). Our analysis was performed on log-transformed RTs, and we included two predictors to measure the VMAC effect (high-low contrast) and the attentional capture effect (AC; low-absent contrasts), a continuous predictor for Block, and the Distractor × Block interaction (either VMAC or AC × Block). We centered the Block predictor and set the hypothesis matrix for the Distractor so that all predictors could be interpreted independently of distractor status. For the RTs analysis, we discarded incorrect responses (acquisition phase: 5.54%; extinction phase: 6.06%) and RTs below 150 ms or above 1800 ms (<0.25% in both phases).

We also analyzed task accuracy in the same way as the RTs analysis. We used general linear mixed models with binomial probability and logit link (Jaeger, 2008) with the same model rationale and structure as the RTs analysis to analyze the likelihood of a participant producing a correct response. We fit all our models with a maximum random effects structure (Barr et al., 2013) supported by the data (Bates et al., 2015; Matuschek et al., 2017). For all models, Satterthwaite-corrected degrees of freedom were used for significance testing.

In the same vein as Watson et al., (2019a) and Garre-Frutos et al. (2024), we also compared the last two blocks of the acquisition phase with the first two blocks of trials of the extinction phase to test whether the VMAC effect reduced once instructions about the extinction were provided. We employed Repeated Measures ANOVAs (RM-ANOVA) with a Singleton factor (High, Low singleton) and a Phase Factor (Acquisition, Extinction) to test whether the VMAC effect changed between phases. We performed this analysis on raw RTs, where all statistical assumptions were met.

To investigate the relationship between VMAC persistence and individual differences, we calculated the average VMAC effect (high vs. low contrast in RTs) for both the Acquisition and Extinction stages separately. We correlated each VMAC effect with the scores of both the UN and UP subscales. We calculated Pearson’s *r* correlation between VMAC in acquisition and extinction with UN and UP in case both variables were normally distributed (Das & Imon, 2016). If any of the measures were not normally distributed, we used Spearman correlations instead. Furthermore, as suggested by studies evaluating the reliability of Experimental measures, data preprocessing decisions that work well for group-level inferences are less optimal for correlational studies (Garre-Frutos et al., 2024; Parsons, 2022; Vadillo et al., 2024). For that reason, for our correlational analysis, we also filtered RTs 3 SDs above or below each participant’s distribution (see Garre-Frutos et al., 2024). Using this filter, we exclude <1.45% of the trials. The split-half reliability^2^ of VMAC in the acquisition stage was *r*_*sb*_ = 0.45, 95% CI [0.27, 0.60], and VMAC in extinction was *r*_*sb*_ = 0.55, 95% CI [0.40, 0.67].

All analyses were performed in R (4.3.1; R core team, 2023). All information about fitted models is presented in the supplementary material, while only the main results are presented in the main text.

## Results

### Acquisition phase

**Fig. 2:**
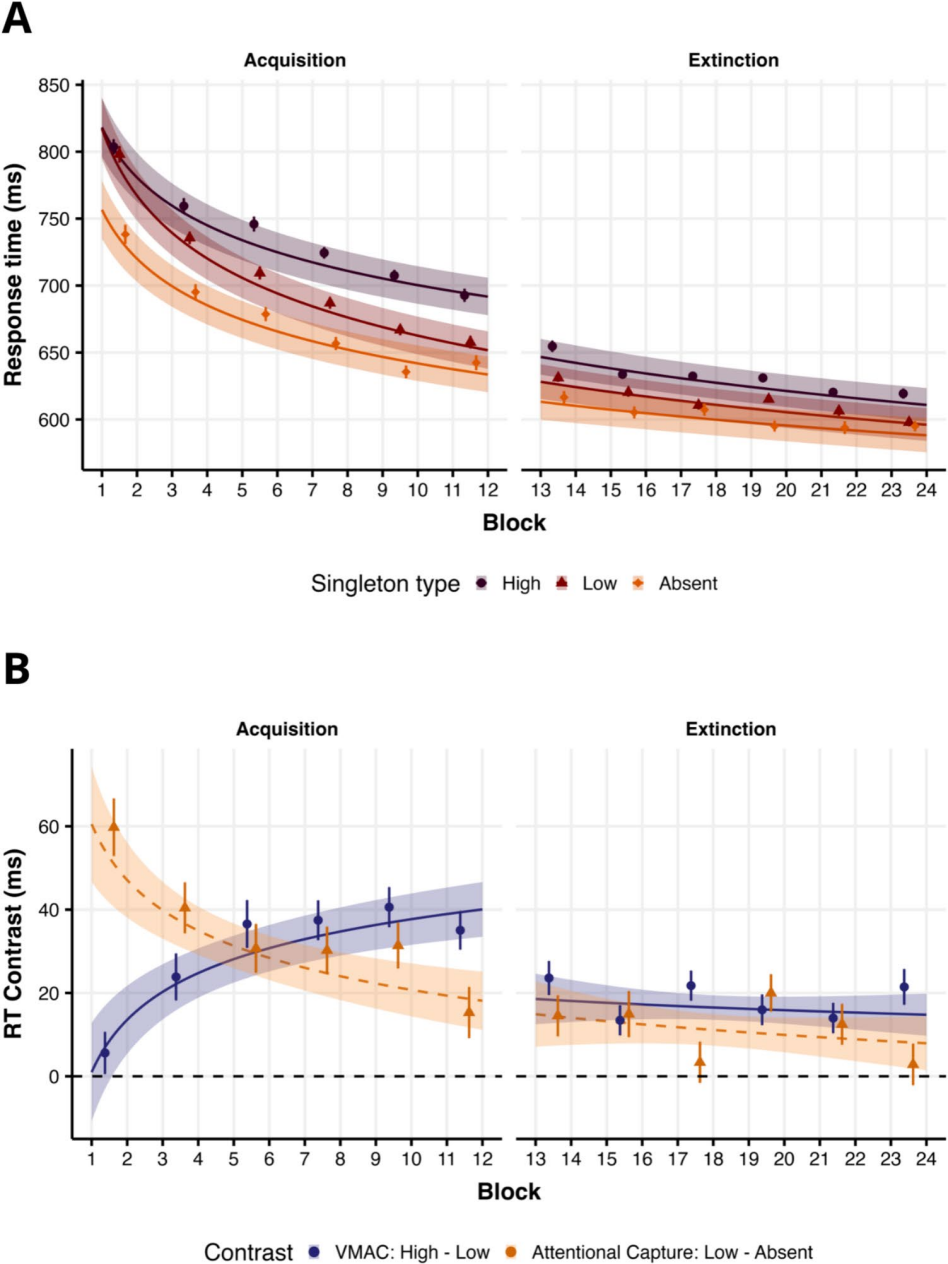
Model predictions for RTs analysis in Experiment 1. **A** Model predictions as a function of singletons across blocks. Lines represent the predicted conditional mean in the response scale, while shaded areas indicate the 95% CI. Raw mean RTs using epochs of 2 blocks are indicated by dots, and error bars represent the standard error of the mean (SEM). **B** Conditional effects, which represent the conditional mean of the high vs. low (VMAC) and low vs. absent (AC) contrasts in the response scale

We analyzed RTs employing a maximum model with random slopes for singleton and block. A power function model was selected due to its lower *AIC* (|Δ_*AIC*_|= 255.6). The model predictions are shown in Fig. 2A. There was a main effect of Block (*β*_*Block*_ = −0.055, *t*(110.1) = −19.74, *p* < 0.001), indicating a general decrease in RTs across the acquisition phase. Second, we found a significant AC effect (*β*_*AC*_ = 0.044, *t*(101.6) = 11.50, *p* < 0.001; *M*_AC_ = 31.1, 95% CI [25.7, 36.4]) with higher RTs in the low-valued distractor condition (*M* = 702, 95% CI[687, 717]) compared to the absent distractor condition (*M* = 672, 95% CI[658, 686]). Third, a significant VMAC effect (*β*_*VMAC*_ = 0.040, *t*(102.7) = 10.05, *p* < 0.001; *M*_VMAC_ = 28.3, 95% CI [22.7, 33.9]) was observed, showing higher RTs for the high-valued singleton (*M* = 731, 95% CI[716, 746]) compared to the low-valued singleton. Additionally, both the AC effect (*β*_*AC x Block*_ = −0.014, *t*(27,530) = −4.05, *p* < 0.001) and the VMAC effect (*β*_*VMAC x Block*_ = 0.017, *t*(27,510) = 6.38, *p* < 0.001) significantly interacted with the block predictor. These interactions are depicted in Fig. 2B, which illustrates that the AC effect diminishes over blocks while the VMAC effect increases.

For accuracy, the maximum model included a random slope for Block. As in previous studies, overall accuracy was near the ceiling (*M* = 0.955, 95% CI[0.948, 0.961]). A significant AC effect (*β*_*AC*_ = −0.277, *z* = −3.54, *p* < 0.001) revealed lower accuracy for the low-valued singleton (*M* = 0.951, 95% CI[0.944, 0.958]) compared to the absent singleton condition (*M* = 0.962, 95% CI[0.956, 0.969]). However, neither Block (*β*_*Block*_ = 0.009, *z* = 0.25, *p* = 0.80), nor VMAC (*β*_*VMAC*_ = −0.009, *z* = −0.17, *p* = 0.86; High-value singleton: *M* = 0.950, 95% CI[0.943, 0.958]) effects were significant. No significant interactions were observed (all *ps* > 0.142).

### Extinction phase

The same analytical approach was applied to the extinction phase. RTs were modeled using an LMM, with the maximum model including a power function model and a random slope for Block (|Δ_*AIC*_|= 4, favoring the power function model). Figure 2A shows the model predictions on the response scale. As in the previous phase, we observed a significant effect of Block (*β*_*Block*_ = −0.016, *t*(120.1) = −8.42, *p* < 0.001), an AC effect (*β*_*AC*_ = 0.018, *t*(27,610) = 5.72, *p* < 0.001; *M*_AC_ = 11.1, 95% CI [7.31, 14.9]), and a VMAC effect (*β*_*VMAC*_ = 0.027, *t*(27,610) = 11.06, *p* < 0.001; *M*_VMAC_ = 16.5, 95% CI [13.58, 19.5]), which reflects higher RTs for the high-value distractor (*M* = 629, 95% CI[617, 641]) compared to low-value distractor (*M* = 613, 95% CI[601, 625]), and lower RTs for the absent distractor condition (*M* = 602, 95% CI[590, 614]) relative to the low-value distractor. Neither the AC x Block interaction (*β*_*AC x Block*_ = −0.003, *t*(27,610) = −1.04, *p* = 0.297), nor the VMAC x Block interaction were significant (*β*_*VMAC x Block*_ = −0.001, *t*(27,620) = −0.60, *p* = 0.56). As illustrated in Fig. 2B, the conditional AC and VMAC effects remained stable across blocks during the extinction phase. We also tested whether the VMAC effect was still present in the last two blocks of trials. A *t*-test revealed that the effect was significant (*M*_high-low_ = 21.45, 95% CI[12.19, 30.71],* t*(102) = 4.60, *p* < 0.001; *d* = 0.45, 95% CI[0.25, 0.65], *BF*_*10*_ = 1278.4) in the last two blocks of the extinction phase, confirming that the VMAC effect was still present at the end of the extinction phase. In the same vein that Watson et al., (2019a) and Garre-Frutos et al. (2024), we also compared the VMAC effect at the end of the acquisition phase (last two blocks) and the beginning of the extinction phase (first two blocks), to test if there is any immediate effect of changing the phase in the VMAC effect. We then run a Repeated Measures ANOVA (RM-ANOVA), with Singleton (High, Low) and Phase (Acquisition, Extinction), showing a significant main effect of Singleton (*F*(1, 102) = 66.57, *MSE* = 1330.21, *p* < 0.001, *η*_*p*_^2^ = 0.395), with higher rates for the high than the low-value distractor, a significant effect of Phase, (*F*(1, 102) = 47.25, *MSE* = 2282.07, *p* < 0.001, *η*_*p*_^2^ = 0.317), reflecting faster RTs on the extinction phase and a significant Singleton x Phase interaction (*F*(1, 102) = 3.99, *MSE* = 845.43, *p* = 0.048, *η*_*p*_^2^ = 0.038). This interaction reflects that the VMAC effect is higher in the acquisition stage (*M*_VMAC_ = 35.04, 95% CI[25.14, 44.94],* t*(102) = 7.02, *p* < 0.001; *d* = 0.69, 95% CI[0.48, 0.91]) compared to the extinction phase (*M*_VMAC_ = 23.60, 95% CI[15.35, 31.86],* t*(102) = 5.67, *p* < 0.001; *d* = 0.45, 95% CI[0.25, 0.65]).

Regarding accuracy, the maximum model included a random slope for singleton. Overall accuracy remained near the ceiling (*M* = 0.950, 95% CI[0.943, 0.957]). Unlike the acquisition phase, no significant AC effect was detected (*β*_*AC*_ = 0.096, *z* = 1.075, *p* = 0.283). Similarly, no significant effects of Block (*β*_*Block*_ = 0.034, *z* = 1.281, *p* = 0.20) or VMAC (*β*_*VMAC*_ = −0.001, *z* = −0.022, *p* = 0.983) were observed. Accuracy rates were very similar across conditions, with high-valued (*M* = 0.952, 95% CI[0.944, 0.959]), low-valued (*M* = 0.952, 95% CI[0.944, 0.959]), and absent singleton distractors (*M* = 0.947, 95% CI[0.938, 0.956]) all showing comparable performance. No significant interactions were observed (all *ps* > 0.227).

### Individual differences

We examined the relationship between the VMAC effect and the tendency to act rashly when experiencing intense negative (NU) and positive (PU) emotions, as measured by the subscales of the S-UPPS-P. Regarding the acquisition phase, neither NU (*r* = 0.006, *p* = 0.952) nor PU (*r* = −0.013, *p* = 0.181) showed significant correlations with the VMAC effect. In the same vein, neither NU (*r* = −0.008, *p* = 0.185) nor PU (*r* = 0.040, *p* = 0.232) was significantly correlated with the VMAC effect during the extinction phase.

## Discussion

In Experiment 1, we entirely replicated the results from Garre-Frutos et al. (2024) and Watson et al., (2019a). The VMAC effect for RTs gradually increased in the acquisition stage, and there was no sign of a reduction in VMAC across the extinction phase. This further suggests that once learned, at least when the task includes both reward and punishment, VMAC is resistant to extinction. Additionally, contrary to our expectations, we did not find any significant correlations between the VMAC in acquisition and extinction stages and our measures of impulsivity (unlike Albertella et al., 2020b, who found a positive relationship between NU and VMAC persistence).

Our inability to find significant associations between the persistence of the VMAC effect and measures of individual differences could be related to several issues (i.e., low-power, high measurement error), including specific procedures employed in Experiment 1. For instance, some authors have suggested that the inclusion of punishments contingent on performance is a less optimal measure of attention sign-tracking (Albertella et al., 2019, 2020a, b). The learning process may change when the high-value distractor predicts high rewards and punishments, introducing processes beyond attentional sign-tracking prompted by cue-reward association, like loss-related sensitivity. In other words, punishments could produce qualitative differences in the learning process underlying VMAC. Another possibility is that participants could change their response strategy, maybe slowing down their responses to increase accuracy and avoid punishments when the high-value distractor is presented. This raises the possibility that when the high-value distractor predicts both high reward and high punishment, punishment may elicit processes not related to the pure effects of feature-rewards associations on attention. The next experiment aimed to shed light on the effect of punishment on VMAC by eliminating it from the task.

## Experiment 2

Experiment 2 was identical to Experiment 1, but we implemented the reward-only variant of the VMAC task (e.g., Albertella et al., 2019, 2020a, b), where participants do not lose points following incorrect responses, and rewards are just omitted.

## Methods

### Participants

Data was collected from 115 volunteers. Four participants were excluded due to data recording failures. The final sample comprised 111 participants (101 females, nine males, and one non-binary), aged 18–30 (*M* = 19.7, *SD* = 2.1). Undergraduate students received course credits for their participation, and the top ten performers in each experiment, based on task performance, received €5 as additional compensation. The present experiment was not formally preregistered.

### Materials

Materials, equipment, and participant recruitment were identical to those used in Experiment 1. Reliability indices for the NU and PU subscales in this sample were Cronbach’s α = 0.80 and α = 0.74, respectively.

#### Value-modulated attentional capture (VMAC) task

In this case, a reward-only variant of the task was used. In both phases, errors resulted in earning 0 points instead of losing points. Otherwise, the task protocol closely followed that of Experiment 1.

### Procedure

The procedure was like that of Experiment 1 except for the VMAC task protocol explained above (punishment omission)^3^.

### Data analysis

For Experiment 2, we followed the same analysis approach as in Experiment 1. In this analysis, we again excluded incorrect responses (acquisition phase: 8.12%; extinction phase: 8.15%) and RTs below 150 ms and above 1800 ms (<0.20% in both phases). For correlational analysis, we additionally removed RTs 3SDs above or below each participant distribution (<1.40% in both phases). The reliability of VMAC during the acquisition stage was *r*_*sb*_ = 0.42, 95% CI [0.22, 0.58], and during the extinction phase was *r*_*sb*_ = 0.46, 95% CI [0.29, 0.60].

## Results

### Acquisition phase

**Fig. 3:**
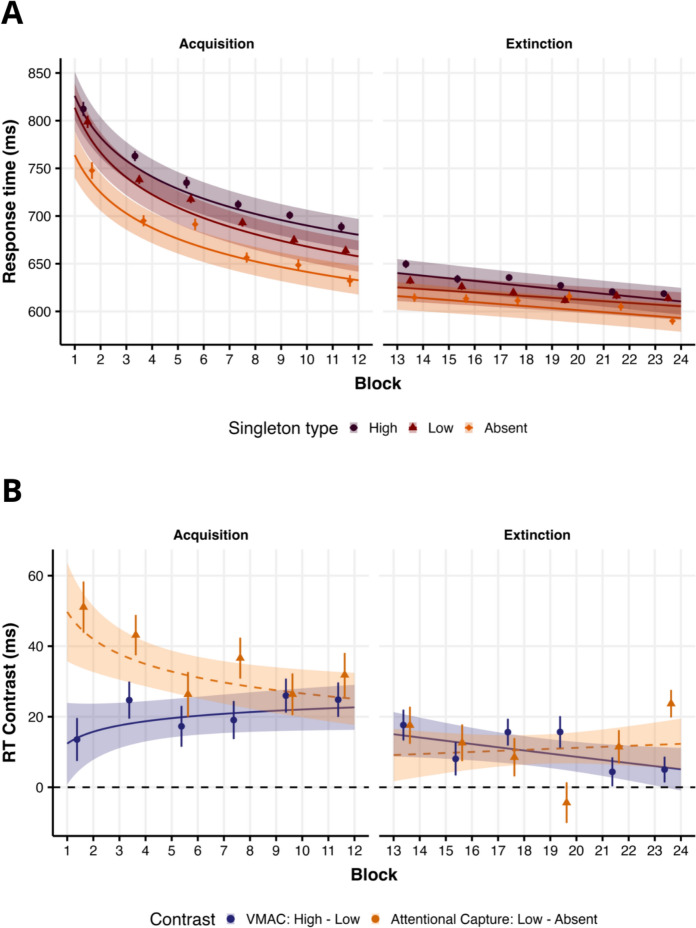
Model predictions for RTs analysis in Experiment 2. **A** Model predictions as a function of singletons across blocks. Lines represent the predicted conditional mean in the response scale, while shaded areas indicate the 95% CI. Raw mean RTs using epochs of 2 blocks are indicated by dots, and error bars represent the SEM. **B** Conditional effects, which represent the conditional mean of the VMAC and AC effect contrasts in the response scale

For the RT analysis, our maximum model included a random effect for Singleton and Block, and a power function model was selected due to its lower *AIC* (|Δ_*AIC*_|= 340.7). The model predictions are shown in Fig. 3A. Our analysis showed a significant effect of Block (*β*_*Block*_ = −0.058, *t*(114.4) = −17.378, *p* < 0.001) due to a general decrease in RTs throughout the acquisition phase, a significant AC effect (*β*_*AC*_ = 0.047, *t*(112.3) = 11.076, *p* < 0.001; *M*_AC_ = 32.6, 95% CI [26.7, 38.6]), with higher RTs for the low-valued distractor (*M* = 705, 95% CI[688, 723]) than for the absent distractor condition (*M* = 673, 95% CI[658, 689]), and a significant VMAC effect (*β*_*VMAC*_ = 0.028, *t*(109.4) = 7.176, *p* < 0.001; *M*_VMAC_ = 19.6, 95% CI [14.2, 25]), with RTs for the high-valued singleton (*M* = 725, 95% CI[708, 743]) being higher than those for the low-valued singleton. Finally, both the AC effect (*β*_*AC*_ _*×*_ _*Block*_ = −0.007, *t*(28,860) = −2.033, *p* = 0.042) and the VMAC effect (*β*_*VMAC*_ _*×*_ _*Block*_ = 0.005, *t*(28,840) = 2.071, *p* = 0.038) interacted significantly with the block predictor. To better visualize this interaction, we plotted the conditional AC and VMAC effect across blocks of trials in Fig. 3B. As can be seen, the AC effect decreases across blocks while the VMAC effect increases.

We first fit the maximum model in the accuracy analysis, including only a random intercept. As in previous studies, the overall accuracy was close to the ceiling (*M* = 0.936, 95% CI[0.927, 0.944]). Regarding the remaining predictors, our analysis revealed a significant effect of Block (*β*_*Block*_ = −0.061, *z* = −1.996, *p* = 0.0459), reflecting a decrease in accuracy across blocks, an AC effect on accuracy (*β*_*AC*_ = −0.16, *z* = −2.532, *p* = 0.013), with accuracy for the low-valued singleton (*M* = 0.936, 95% CI[0.927, 0.945]) being lower than for the absent singleton condition (*M* = 0.945, 95% CI[0.936, 0.954]), and also a significant VMAC effect on accuracy (*β*_*VMAC*_ = −0.134, *z* = −2.996, *p* = 0.003), due to a decrease in accuracy when the high-valued singleton appeared in the display (*M* = 0.945, 95% CI[0.927, 0.954]) compared to the low-value distractor. There were no other significant effects or interactions (all *ps* > 0.641).

### Extinction phase

The same analysis was performed for the extinction phase. As in the previous phase, the RTs were submitted to LMM, where the maximum model structure was again a model with a random slope for singletons and a block. Unlike the previous model, we chose a linear model instead of a power function (|Δ_*AIC*_|= 12 in favor of the linear model). Figure 3A shows the model predictions in the response scale. As in the previous phase, we observed a significant effect of Block (*β*_*Block*_ = −0.012, *t*(119.7) = −5.711, *p* < 0.001), an AC effect (*β*_*AC*_ = 0.018, *t*(111.2) = 5.115, *p* < 0.001; *M*_AC_ = 10.8, 95% CI [6.62, 14.9]), and a VMAC effect (*β*_*VMAC*_ = 0.016, *t*(109.5) = 4.801, *p* < 0.001; *M*_VMAC_ = 10.1, 95% CI [5.98, 14.1]), which reflects higher RTs for the high (*M* = 627, 95% CI[614, 641]) than the low-value distractor (*M* = 617, 95% CI[604, 631]), and lower RTs for the absent distractor condition (*M* = 607, 95% CI[594, 620]) compared to the low-value distractor. Interestingly, while we did not observe an AC × Block interaction (*β*_*AC*_ _*×*_ _*Block*_ = 0.002, *t*(28,940) = 0.580, *p* = 0.562), the VMAC × Block interaction was significant (*β*_*VMAC*_ _*×*_ _*Block*_ = −0.005, *t*(28,910) = −17.378, *p* = 0.044). As shown in Fig. 3B, while the conditional AC effect remained constant across blocks, the VMAC effect decreased until it disappeared at the end of the extinction phase. As in the previous experiment, we also tested whether the effect was still present in the last two blocks of trials. A t-test revealed that the effect was not significant (*M*_high-low_ = 5.01, 95% CI[−2.62, 12.66],* t*(110) = 1.30, *p* = 0.196; *d* = 0.12, 95% CI[−0.06, 0.32]) in the last two blocks of the extinction phase, confirming that the VMAC effect was not present at the end of this phase. Lastly, as in Experiment 1, we compared whether the VMAC effect changes between phases just after giving the extinction instruction. An RM-ANOVA revealed a main effect of Singleton (*F*(1, 110) = 32.35, *MSE* = 1546.88, *p* < 0.001, *η*_*p*_^2^ = 0.227), indicating a significant VMAC effect, and effect of Phase (*F*(1, 110) = 82.51, *MSE* = 1684.16, *p* < 0.001, *η*_*p*_^2^ = 0.429), with faster RTs for the extinction phase, and no Singleton × Phase interaction (*F*(1, 110) = 1.13, *MSE* = 1272.39, 0.91, *p* = 0.290, *η*_*p*_^2^ = 0.010), reflecting no significant reduction between the acquisition (*M*_VMAC_ = 24.83, 95% CI[14.75, 34.90],* t*(110) = 4.88, *p* < 0.001; *d* = 0.47, 95% CI[0.27, 0.66]) and extinction (*M*_VMAC_ = 17.63, 95% CI[7.73, 27.53],* t*(110) = 3.532, *p* < 0.001; *d* = 0.34, 95% CI[0.14, 0.53]) phases.

Regarding accuracy, the maximum model included only the random intercept for participants. Overall task accuracy was also high but lower than in Experiment 1(*M* = 0.932, 95% CI[0.923, 0.94]). In contrast to the acquisition phase, no significant AC (*β*_*AC*_ = −0.071, *z* = −1.147, *p* = 0.252) or VMAC (*β*_*VMAC*_ = −0.557, *z* = −1.23, *p* = 0.217) effects on accuracy were found. In other words, general task accuracy was largely the same for high-valued (*M* = 0.928, 95% CI[0.919, 0.937]), low-valued (*M* = 0.932, 95% CI[0.923, 0.941]), and absent (*M* = 0.936, 95% CI[0.926, 0.946]) singleton distractor conditions. Interestingly, there was a significant VMAC × Block interaction (*β*_*VMAC*_ _*×*_ _*Block*_ = −0.134, *z* = −3.00, *p* = 0.003). The decrease in task accuracy for the high-value singleton we observed in the acquisition phase gradually disappears during the extinction phase (see Fig. 3B), a result that converges with the decline in the VMAC effect on RTs in the extinction phase. No other effect or interaction was significant (*ps* > 0.138).

### Individual differences

As in Experiment 1, we examined the relationship between the VMAC effect and the NU and PU subscales. No significant correlations were found during the acquisition phase (NU: *r* = −0.050, *p* = 0.606; PU: *r* = 0.045, *p* = 0.641). In the extinction phase, a significant positive correlation was observed between the persistence of the VMAC effect and the PU subscale (*r* = 0.238, *p* = 0.012), as expected, while no significant correlation was found with the NU subscale (*r* = 0.097, *p* = 0.310).

## Discussion

In Experiment 2, we replicated the results of Experiment 1 regarding the acquisition of the VMAC effect in terms of RTs. Unlike the previous experiment, we also found a gradual extinction of the VMAC effect across the extinction stage, with no VMAC effect in the last two blocks. Interestingly, in contrast with previous studies applying both reward and punishment contingent to performance, we observed significant differences between distractor conditions in accuracy (see also Albertella et al., 2019), with both AC and VMAC effects in the learning stage. Critically, we also observed an extinction effect of VMAC in accuracy, with a gradual decrease in the interference caused by the high-valued distractor compared to the low-valued distractor, which converges with the decrease in the VMAC effect observed in RTs during this same stage.

In Experiment 1, we failed to observe the extinction of the VMAC effect, suggesting that the learned attentional bias is resistant to Pavlovian extinction (DeTommaso & Turatto, 2021; Garre-Frutos et al., 2024; Le et al., 2025; Watson et al., 2019a). In Experiment 2, on the contrary, we observed the same pattern of results in both RTs and accuracy during the second stage, a gradual reduction in the previously acquired attentional bias. A potential explanation for this difference is that punishments affect not only the learning process of VMAC but also the response strategies employed by participants (i.e., speed-accuracy tradeoff). In other words, the reduction observed during the extinction stage in the learned attentional bias could be explained by qualitative differences in learning (e.g., introducing punishments changes the nature of the task, undermining the parallelism with the phenomenon of sign-tracking which would be based on cue-reward association only) or by a lingering response strategy applied during the acquisition stage that is transferred to the extinction phase. In other words, it could be that in the extinction stage of Experiment 1, participants continue to be cautious in the presence of the high-value distractor even if there are no incentives to maintain the previous response strategy with the previously high-value distractor. If the latter is true, we expect to find an overall difference in RTs and accuracy between experiments, with participants being slower and more accurate in Experiment 1 than in Experiment 2 for both phases.

## Between experiment comparison

To further explore the observed pattern of results, we directly compared Experiments 1 and 2 to test the effect of punishments on RTs and accuracy during the acquisition and extinction stages. To that end, we added Experiment as a new predictor using deviation coding and independently ran the above-described models on the acquisition and extinction stages. To simplify the analysis, we excluded absent trials so that we could only test how both experiments differed regarding the VMAC effect.

### Acquisition phase

**Fig. 4:**
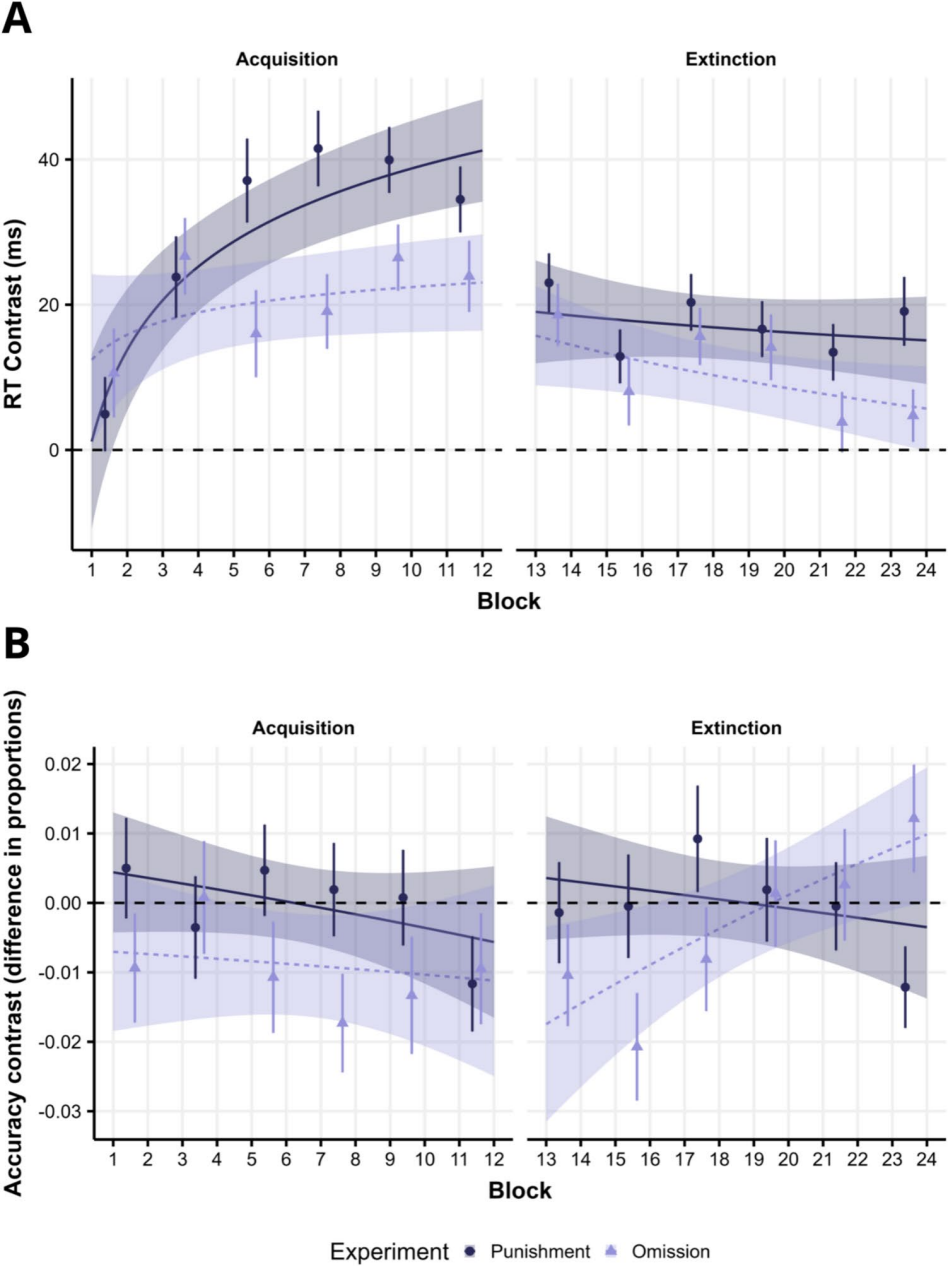
Comparisons of the VMAC effect in Experiments 1 (Punishment) and 2 (Omission). Conditional VMAC effect for the model comparing Experiments 1 (incorrect responses led to punishments) and 2 (incorrect responses result in reward omission) for RTs (**A**) and accuracy (**B**). As in previous Figures, lines represent the model conditional VMAC effect, shaded areas the 95% CI. Dots depict raw data (Epochs of 2 blocks), and error bars represent the SEM

RT analysis for the acquisition phase included a random slope for Block and Singleton. Regarding the main effects and interactions with the Experiment predictor, we found a significant VMAC × Experiment interaction (*β*_*VMAC*_ _*×*_ _*Experiment*_ = 0.013, *t*(212.1) = 2.29, *p* = 0.023), showing that the VMAC effect was stronger in Experiment 1 compared to Experiment 2. Additionally, we found a significant three-way interaction (*β*_*VMAC*_ _*×*_ _*Block*_ _*×*_ _*Experiment*_ = 0.011, *t*(46,880) = 3.00, *p* = 0.003), representing that the VMAC effect increases more across blocks in Experiment 1 compared to Experiment 2 (see Fig. 4A, left). There were no other significant main effects or interactions with the Experiment predictor (*ps* > 0.683).

For accuracy, we again fitted a model with a random slope for Block and Singleton, showing a significant effect of Experiment (*β*_*Experiment*_ = 0.355, *z* = 3.47, *p* < 0.001), with participants showing an overall higher accuracy in Experiment 1 than in Experiment 2. On the other hand, the VMAC × Experiment interaction (*β*_*VMAC*_ _*×*_ _*Experiment*_ = 0.127, *z* = 1.72, *p* = 0.085) was non-significant. Nevertheless, there seems to be a nominal tendency to show lower accuracy in Experiment 2 for the high-value compared to the low-value distractor than in Experiment 1 (Fig. 4B). There were no more significant interactions with the Experiment predictor (*ps* > 0.171).

### Extinction phase

For the RT analysis in the extinction phase, we fitted a model with random slopes for Block and Singleton. We found a significant VMAC × Experiment interaction (*β*_*VMAC*_ _*×*_ _*Experiment*_ = 0.011, *t*(210.8) = 2.20, *p* = 0.029), again representing a higher VMAC effect in Experiment 1 than in Experiment 2. Although the three-way interaction was not significant in this model (*β*_*VMAC*_ _*×*_ _*Experiment*_ = 0.003, *t*(46,990) = 0.96, *p* = 0.337), critically, if we compare the last two blocks of the extinction phase between Experiments using an RM ANOVA, we found a significant difference in VMAC between experiments (*F*(1, 212) = 7.46, *MSE* = 967.68, *p* = 0.007, *η*_*p*_^*2*^ = 0.034). As depicted in Fig. 4A (right), the reduction in VMAC across blocks in Experiment 2 is very gradual, and only at the end of the extinction phase is it completely reduced. Even though both Experiments 1 and 2 had an exceptionally high sample size (*N* = 214), thousands of participants may be needed for this kind of interaction to have enough statistical power (see Brysbaert, 2019). There were no other significant interactions with the Experiment predictor (*ps* > 0.159).

Regarding accuracy for the extinction phase, we fitted a GLMM with a random slope for Singleton. Again, we observed significant differences in accuracy between Experiments (*β*_*VMAC*_ = −0.134, *z* = −2.996, *p* = 0.003), with higher accuracy in Experiment 1 than in Experiment 2. Interestingly, we found a significant three-way interaction (*β*_*VMAC*_ = −0.134, *z* = −2.996, *p* = 0.003), reflecting a reduction in the VMAC effect for accuracy across blocks only for Experiment 2 (see Fig. 4B, right). There were no other significant effects or interactions (*ps* > 0.576).

### SAT analysis

**Fig. 5:**
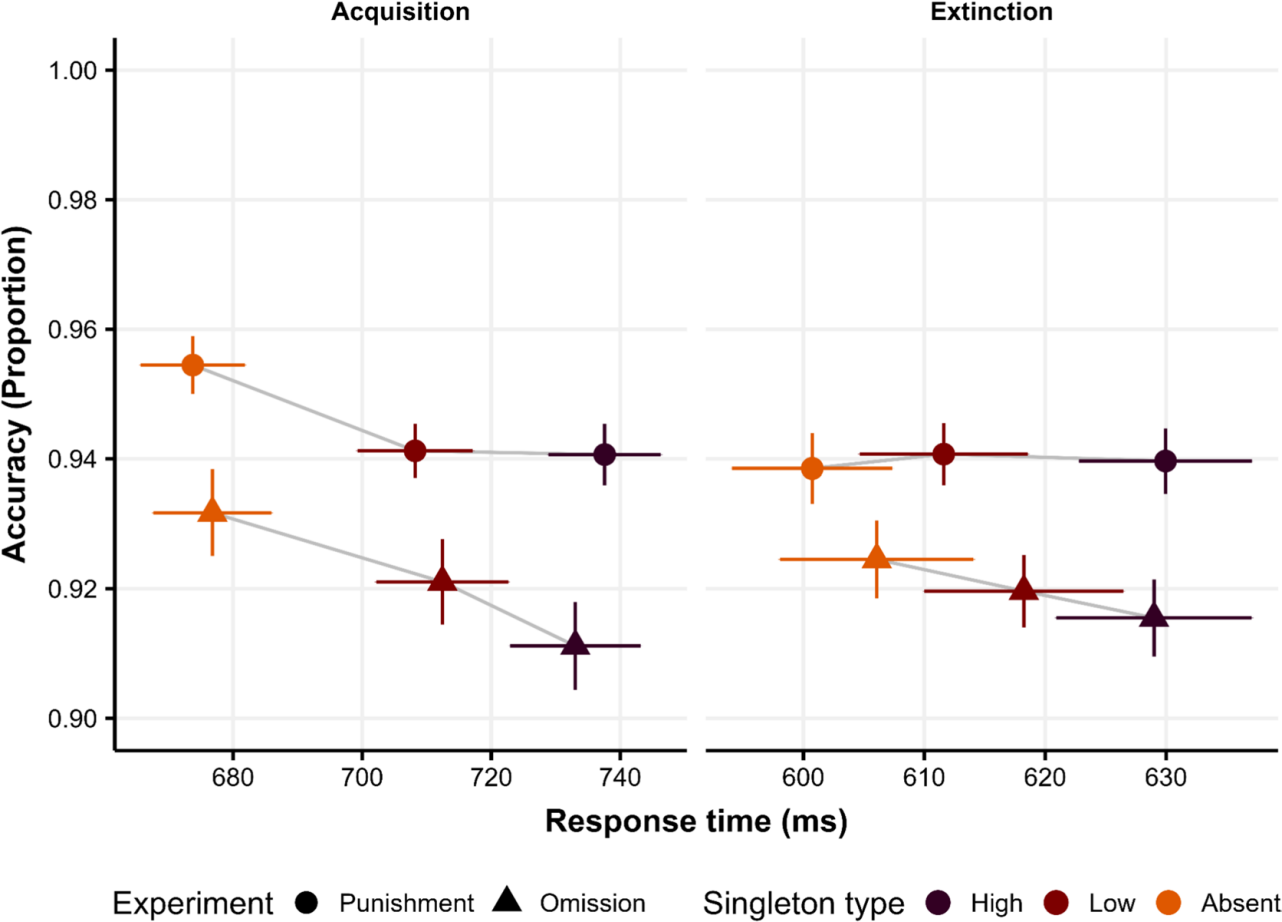
An SATF showing the tradeoff between accuracy and RTs. The shape of the dots represents the Experiment (Experiment 1: Punishments; Experiment 2: Omission) and colors the singleton distractor condition. Error bars show SEM

As hypothesized, participants may adjust their response strategy when they perceive that the high-value distractor would also be accompanied by high punishments when incorrect responses are possible. Our previous results comparing experiments indeed indicate evidence of SAT in the acquisition stage. To gather evidence for the previous, we visualize the relationship between accuracy and RTs using an SAT Function (SATF; Heitz, 2014). The SATF visualization consists of representing the proportion of correct responses as a function of the mean of the RT for each condition. In Fig. 5, we show the SATF for each distractor condition (also including absent distractor condition), Experiment (Experiments 1 and 2), and the experimental stage as well (Acquisition, Extinction).

Due to practice, participants are faster in the Extinction phase than in the Acquisition phase, and overall accuracy is higher in Experiment 1 (Punishments) than in Experiment 2 (Omission). As can be seen, overall, there is no appreciable SAT because Singleton distractor conditions with lower accuracy also show higher RT. Interestingly, in Experiment 2, there is a clear pattern where participants are both slower and less accurate with the high-value distractor, followed by the low-value distractor. This effect is reduced in the extinction stage, but the same pattern is nominally appreciable. On the contrary, there is no interference from the high compared to the low-value distractor in Experiment 1, which suggests the presence of SAT between Experiments.

### Individual differences

To examine whether the observed correlations between the VMAC effect (acquisition and extinction phases) and the NU and PU scores differed significantly between Experiments 1 and 2, we applied the two-tailed Fisher’s r-to-z transformation. The analyses comparing experiments revealed no significant differences between any of the correlations (see Table S7).

## General discussion

In the present two experiments, we aimed to study the effect of Pavlovian extinction on the VMAC effect as well as the effect of punishments over VMAC. In Experiment 1, we mostly replicated the results of Garre-Frutos et al. (2024) during the acquisition and the extinction stage; this time, instead of completely omitting rewards, the value of the previously high-value stimulus was equated to that of the low-value. With this procedure, we replicated previous results, showing that the VMAC effect increases progressively across the acquisition stage (see also Garre-Frutos et al., 2024), and did not decrease across the extinction stage. In Experiment 2, we mostly replicated the procedure of Experiment 1, but we employed a version of the task where participants did not lose points after incorrect responses (Albertella et al., 2019, 2020a, b) in either stage. With this procedure, we replicated the results of Experiment 1 in the acquisition stage, but we also showed a significant VMAC effect in accuracy. In the extinction phase, we found a significant reduction in the VMAC effect across blocks and no VMAC effect at the end of the extinction phase. Similarly, we found a significant reduction in the VMAC effect found in accuracy across trials, converging with the same result on RTs. Regarding individual differences, in Experiment 1, we did not find any significant association between the persistence of VMAC and our measures of affect-driven impulsivity, while in Experiment 2, we observed a significant association with one of these measures of positive urgency. Finally, a comparison between Experiments 1 and 2 revealed that VMAC for Experiment 1 was significantly larger than in Experiment 2 in RTs, but compared to Experiment 2, there were no effects in accuracy. In the Extinction stage, the VMAC effect was only reliably observed at the end of the Extinction stage in Experiment 1, and critically, there was a significant difference in the reduction in the interference generated by the high-value distractor between Experiments.

As we review above, cues that have the potential to predict significant outcomes in the environment often also gain motivational salience (Berridge et al., 2009; Flagel & Robinson, 2017; Flagel et al., 2011), both at the response and the attentional level (Colaizzi et al., 2020). Specifically, cues that predict reward gain increased attentional priority, but cues that predict negative outcomes also have an increased capacity to capture attention (Watson et al., 2019b). Some studies have shown that cues associated with significant aversive outcomes, such as electric shocks, receive higher attentional priority (Mikhael et al., 2021; Nissens et al., 2017). Other studies have found that monetary losses also produce comparable effects to reward gains (Le Pelley et al. 2019b; Müller et al., 2016; Wentura et al., 2014). In those studies, a punishment-predictive cue indicates that participants may lose reward when such cues are presented as a distractor in visual search tasks, but participants can avoid losing money if they give a fast and accurate response. As suggested by Becker et al. (2020), distractors associated with high monetary loss, when participants can avoid punishment, may be perceived as more rewarding, which may introduce a confound. When participants cannot avoid punishments in any way, other studies have failed to find attentional capture by monetary loss (Becker et al., 2020; Bucker & Theeuwes, 2016; Freichel et al., 2023). Although previous research has shown that monetary loss is often not enough to observe VMAC, it does not mean that a cue that predicts both a high reward and high monetary loss is completely equal to a cue that only predicts a high reward. This has been suggested by several authors employing VMAC as a measure of attentional sign-tracking in individual differences studies (Albertella et al., 2019, 2020a, b), using the so-called *reward-only* variant of the VMAC task. Using the reward-only version of the task, in Experiment 2, we have shown that VMAC affects both RTs and accuracy during acquisition compared to Experiment 1 (rewards and punishments). Even if the effect size of VMAC in RTs is higher in Experiment 1 during the acquisition stage, the VMAC effect in accuracy is absent, suggesting that, comparing between experiments, punishment eliminates the effect on accuracy by increasing RTs and reducing errors produced by the high-value distractor. In other words, our results suggest a subtle tradeoff between RTs and accuracy.

It is widely known that the use of reward or payoff significantly influences response strategies employed by participants (for a review see Heitz, 2014). Consequently, a potential effect of making the high-value distractor a predictor of both rewards and punishments is the impact on participants’ response strategies. In this vein, the positive effect of punishment on accuracy in Experiment 1, and the lack of an extinction effect in RTs in this case, may be related to a participants’ strategy by which response is slowed down during acquisition to reduce the probability of point’s loss, thus reducing errors and overshadowing the interference caused by the high-value distractor in accuracy. This strategy may continue during the second stage, thus maintaining the effect throughout. We detected a higher VMAC effect in Experiment 1 during extinction compared to Experiment 2, and again, the SATF (Fig. 5) shows that the same pattern observed in accuracy is appreciable between phases in both Experiment 1 and Experiment 2, strongly suggesting that the inclusion of punishment is effectively affecting response strategies.

The previous possibility has broader implications. The use of the VMAC effect as a measure of attentional sign-tracking could be compromised if part of the variability in the effect does reflect another construct. As Garre-Frutos et al. (2024) showed, internal consistency in some data preprocessing specifications to calculate the VMAC effect was above the typical threshold recommended for individual difference research (Nunnally, 1978). Nevertheless, part of the “true” variability of the VMAC effect may be contaminated by other processes, such as participants’ response strategies. Congruently, we could only replicate one of the results previously found by Albertella et al. (2019) with the reward-only version of the task. However, we failed to replicate the association between VMAC persistence and individual differences in NU, which could be explained both because our study may have been underpowered to detect the correlations usually found in individual differences studies with VMAC, and because of the low reliability of VMAC in the present studies; as a result, our target correlations may be severely attenuated. Although the internal consistency of VMAC is often considered acceptable (Garre-Frutos et al., 2024; Kim et al., 2025; Watson et al., 2024), test–retest reliability is extremely low (Anderson & Kim, 2019; Freichel et al., 2023), which makes possible that although there are true variations in VMAC beyond trial-by-trial variability (Rouder & Haaf, 2019), it cannot be considered a stable measure or “trait-like” (see Anderson et al., 2020). In any case, as suggested by other researchers, the reward-only variant of the task may provide a purer measure of incentive salience as attentional sign-tracking, based on a cue-reward Pavlovian association, which, eventually, should extinguish. Results regarding the extinction stage add to this possibility. VMAC measured both in terms of RTs and accuracy disappears at the end of this stage in Experiment 2, while the effect in RTs observed in Experiment 1 persists during the second stage, as previously reported in studies using the ‘mixed’ variant of the task (rewards and punishments).

Although using only reward may increase the likelihood that VMAC scores truly reflect variability in sign-tracking propensity, the VMAC task developed by Le Pelley et al. (2015) also includes other features not directly related to the phenomenon of sign-tracking. Perhaps the most important aspect of this task is the fact that participants attend to distracting stimuli even when aware that this is counterproductive to their instrumental goals (Pearson et al., 2015). However, this also implies an instrumental relationship between distractors and outcomes, which might account for part of the variability observed in VMAC scores, potentially modulating the participants’ efforts to control distraction. A clear illustration of the impact of this instrumental relationship can be found in the study by Pearson and Le Pelley (2020). In this study, one group of participants performed the VMAC task under typical conditions, where looking at the distractor before the target eliminated a potential reward. Critically, another group underwent the same task, but their reward outcomes were yoked to the responses of the first group—meaning that their reward and omission patterns precisely mirrored those experienced by participants in the first group. With this manipulation, Pearson and Le Pelley showed that when reward administration was completely response-independent, the VMAC effect significantly increased (see also Pearson & Le Pelley, 2021). As recently suggested by other authors, this feature of response independence may be particularly relevant for measuring sign-tracking (Basel & Lazarov, 2023; Colaizzi et al., 2020; Heck et al., 2025). Therefore, we propose that designs in which the reward is completely response-independent (Bucker & Theeuwes, 2017; Pearson & Le Pelley, 2020) might be even better suited for assessing attentional sign-tracking.

Another important implication of the role of rewards and punishments in VMAC is how both could impact its learning process. As discussed above, observing Pavlovian extinction serves as evidence to validate the formation of an association. Some authors have suggested that VMAC is consistent with the principle proposed by Mackintosh (1975), which postulates that learning should be faster for cues that serve as good predictors of significant outcomes, a phenomenon that has been reported in the Pavlovian learning literature in both animals (Ward et al., 2012) and humans (Livesey & McLaren, 2007). Subsequent research showed that the same principle applies to attention (Le Pelley et al., 2011). Although currently, there is no formal model on how VMAC is learned and influences attentional priority, Le Pelley et al. (2016) proposed a formulation of the Mackintosh model of Pavlovian conditioning^4^ in which the attention received by a cue is a direct function of its absolute associative strength. This model can, in principle, predict the acquisition of the VMAC effect (see also Jeong et al., 2023). However, recent research has found that when a distractor is associated with higher reward variability, it also receives increased attentional priority (Cho & Cho, 2021; Le Pelley et al., 2019a, b; Pearson et al., 2024). This uncertainty-driven enhancement has also been linked to sign-tracking in animals (Anselme et al., 2013; Hellberg et al., 2018), where reward uncertainty significantly increases incentive salience attributed to predictive cues, amplifying approach and engagement behaviors typically observed in gambling-like contexts. The effects of uncertainty on attention is believed to reflect the principle proposed by Pearce and Hall (1980) and has been operationalized as outcome variance (Pearson et al., 2024). In other words, cues associated with higher outcome variance are associated with higher prediction errors (i.e., a discrepancy between expectations and actual perceived outcome), which ultimately increase the attentional priority of such cues. In studies employing the present paradigm, reward magnitude is the key variable manipulated, but outcome variance is not controlled. This raises the possibility that punishments, defined as reward loss, may affect overall outcome variance, thus qualitatively altering learning. If this is correct, the present pattern of result may indicate that increasing outcome variability is making VMAC more difficult to extinguish because the processes by which the distractor signaling high reward gains incentive salience is qualitatively different. In any case, there is little understanding of how value (Mackintosh principle) and uncertainty (Pearce-Hall principle) interact to influence attentional priority, and more research is needed to elucidate if mixing rewards and punishments may affect both.

In summary, our results suggest that mixing monetary gains and losses can impact the response strategies and, possibly, the learning process measured by VMAC. If researchers want to validate VMAC as a measure of attentional sign-tracking, we recommend avoiding employing punishments contingent on performance. Nevertheless, further research is needed to improve the validity of VMAC as a measure of attentional sign-tracking.

## Supplementary Information

Below is the link to the electronic supplementary material.

Supplementary file 1 (DOCX 82 KB)

## Data Availability

All data, materials, and analysis scripts related to the present study are publicly available at https://osf.io/pezvg/, and also in the following GitHub repository: https://github.com/franfrutos/vmac_extinction. The main text reports all data exclusions, manipulations, and measurements. This study was not formally preregistered before data collection.

## Footnotes

1 Note that a pure definition of Pavlovian extinction typically involves the presentation of the conditioned stimuli alone (without any unconditioned stimuli). Our extinction procedure may resemble the idea of ‘successive negative contrasts’ (Phelps et al., 2015). We believe this approach may be better suited to test the extinction of VMAC, given that what participants learn is that the distractor is relatively more valuable than others (Kim & Beck, 2020). This procedure more closely mirrors that of Pavlovian extinction, where only the relationship between the cue and reward for the to-be-extinguished stimulus is usually modified, leaving the rest of the stimuli unaffected.

2 Following previous work from our lab (Garre-Frutos et al., 2024), we computed the split-half reliability of VMAC following a random permutation approach (Parsons et al., 2019). This method involves randomly splitting trials and calculating the correlation between the VMAC effect in each split. The previous procedure was repeated 10,000 times to get a distribution of split-half correlations. Then we applied the spearman-brown prophecy formula (Spearman, 1910) to correct for the reduced number of trials due to splitting. We report the mean and the 95% bootstrapped CIs of the spearman-brown corrected reliabilities (*r*_sb_).

3 As part of a broader study, participants in Experiment 2 completed a Pavlovian to instrumental transfer task (Hinojosa-Aguayo & González, 2020) in the same session, either before or after the VMAC task, random order, with a 10-min resting period between tasks. To confirm that task order did not influence the present result, we re-analyzed results from Experiment 2, showing that task order did not interact with any predictor during Acquisition (all *ps* > .284) or Extinction stages (all *ps* > .302) in the fitted models.

4 Mackintosh never proposed an explicit formulation of his idea. Le Pelley et al. (2016) based its model on a prior formulation in Le Pelley (2004).
