# Supplementary material for: The effect of reward and punishment on the extinction of attentional capture elicited by value-related stimuli
Source: Psychol Res. 2025 Apr 16;89(3):89. doi: 10.1007/s00426-025-02115-2 (PMC12003608; doi:10.1007/s00426-025-02115-2)
Supplement: Supplementary file 1 — (DOCX 82 KB) [file 426_2025_2115_MOESM1_ESM.docx]

# Supplementary Materials to accompany

**The effect of reward and punishment on the extinction of attentional capture elicited by value-related stimuli**

Francisco Garre-Frutos^1,2^, Adriana Ariza^1,2^, and Felisa González^1,2^

^1^Mind, Brain, and Behavior Research Center (CIMCYC), University of Granada, Granada, Spain

^2^ Department of Experimental Psychology, University of Granada, Granada, Spain

##

## Tables for LMMs and correlations

**Table S1**

*Model summaries for the selected models of RTs and accuracy during the acquisition phase in Experiment 1.*

|  | **RTs** | | | Accuracy | | |
| --- | --- | --- | --- | --- | --- | --- |
| *Predictors* | *Estimates* | *CI* | *p* | *Odds Ratios* | *CI* | *p* |
| (Intercept) | 6.532 | 6.512 – 6.553 | **<0.001** | 21.220 | 18.390 – 24.485 | **<0.001** |
| VMAC | 0.040 | 0.033 – 0.048 | **<0.001** | 0.991 | 0.891 – 1.102 | 0.863 |
| AC | 0.044 | 0.037 – 0.052 | **<0.001** | 0.758 | 0.650 – 0.884 | **<0.001** |
| Block | -0.055 | -0.061 to -0.050 | **<0.001** | 1.009 | 0.939 – 1.086 | 0.801 |
| VMAC × Block | 0.017 | 0.012 – 0.022 | **<0.001** | 0.924 | 0.831 – 1.027 | 0.142 |
| AC × Block | -0.014 | -0.021 to -0.007 | **<0.001** | 0.957 | 0.821 – 1.115 | 0.571 |
| **Random Effects** | | | | | | |
| σ^2^ | 0.0408 | | | 3.290 | | |
| τ_00_ | 0.0108 _Intercept_ | | | 0.432 _Intercept_ | | |
| τ_11_ | 0.0009 _VMAC_ | | |  | | |
|  | 0.0003 _AC_ | | |  | | |
|  | 0.0006 _Block_ | | | 0.028 _Block_ | | |
| ρ_01_ | -0.26 | | | -0.11 _Block_ | | |
|  | 0.53 | | |  | | |
|  | -0.24 | | |  | | |
| ICC | 0.236 | | | 0.12 | | |
| N | 103 | | | 103 | | |
| Observations | 27,896 |  |  | 3,708 |  |  |
| Marginal R^2^ / Conditional R^2^ | 0.073 / 0.282 | | | 0.005 / 0.128 | | |

*Note*. Bold entries denote statistical significance. *p*-values were computed using Satterwhite correction. CI = Confidence interval; ICC = Intraclass correlation coefficient. τ = Random effects, ρ = correlation between random effects.

**Table S2**

*Model summaries for the selected models of RTs and accuracy during the extinction phase in Experiment 1.*

|  | **RTs** | | | Accuracy | | |
| --- | --- | --- | --- | --- | --- | --- |
| *Predictors* | *Estimates* | *CI* | *p* | *Odds Ratios* | *CI* | *p* |
| (Intercept) | 6.400 | 6.381 – 6.419 | **<0.001** | 19.057 | 16.504 – 22.005 | **<0.001** |
| VMAC | 0.027 | 0.022 – 0.031 | **<0.001** | 0.999 | 0.873 – 1.142 | 0.983 |
| AC | 0.018 | 0.012 – 0.024 | **<0.001** | 1.101 | 0.924 – 1.313 | 0.283 |
| Block | -0.016 | -0.019 to -0.012 | **<0.001** | 1.035 | 0.982 – 1.091 | 0.200 |
| VMAC × Block | -0.001 | -0.006 – 0.003 | 0.548 | 0.953 | 0.857 – 1.059 | 0.373 |
| AC × Block | -0.003 | -0.010 – 0.003 | 0.297 | 0.918 | 0.799 – 1.055 | 0.227 |
| **Random Effects** | | | | | | |
| σ^2^ | 0.0335 | | | 3.290 | | |
| τ_00_ | 0.0097 _Intercept_ | | | 0.447 _Intercept_ | | |
| τ_11_ |  | | | 0.037 _VMAC_ | | |
|  |  | | | 0.112 _AC_ | | |
|  | 0.0002 _Block_ | | |  | | |
| ρ_01_ | 0.04 | | | 0.13 _VMAC_ | | |
|  |  | | | 0.44 _AC_ | | |
| ICC | 0.228 | | | 0.13 | | |
| N | 103 | | | 103 | | |
| Observations | 27,819 |  |  | 3,708 |  |  |
| Marginal R^2^ / Conditional R^2^ | 0.013 / 0.235 | | | 0.002 / 0.129 | | |

*Note*. Bold entries denote statistical significance. *p*-values were computed using Satterwhite correction. CI = Confidence interval; ICC = Intraclass correlation coefficient. τ = Random effects, ρ = correlation between random effects.

**Table S3**

*Model summaries for the selected models of RTs and accuracy during the acquisition phase in Experiment 2.*

|  | **RTs** | | | Accuracy | | |
| --- | --- | --- | --- | --- | --- | --- |
| *Predictors* | *Estimates* | *CI* | *p* | *Odds Ratios* | *CI* | *p* |
| (Intercept) | 6.531 | 6.508 – 6.555 | **<0.001** | 14.696 | 12.731 – 16.965 | **<0.001** |
| VMAC | 0.028 | 0.020 – 0.035 | **<0.001** | 0.875 | 0.801 – 0.956 | **0.003** |
| AC | 0.047 | 0.039 – 0.055 | **<0.001** | 0.850 | 0.750 – 0.964 | **0.011** |
| Block | -0.058 | -0.064 to -0.051 | **<0.001** | 0.941 | 0.886 – 0.999 | **0.046** |
| VMAC × Block | 0.005 | 0.000 – 0.011 | **0.038** | 0.979 | 0.896 – 1.070 | 0.643 |
| AC × Block | -0.007 | -0.014 to -0.000 | **0.042** | 0.971 | 0.856 – 1.100 | 0.641 |
| **Random Effects** | | | | | | |
| σ^2^ | 0.0422 | | | 3.290 | | |
| τ_00_ | 0.0155 _Intercept_ | | | 0.512 _Intercept_ | | |
| τ_11_ | 0.0009 _VMAC_ | | |  | | |
|  | 0.0007 _AC_ | | |  | | |
|  | 0.0010 _Block_ | | | 0.022 _Block_ | | |
| ρ_01_ | -0.13 | | | 0.010 _Block_ | | |
|  | 0.44 | | |  | | |
|  | -0.16 | | |  | | |
| ICC | 0.299 | | | 0.14 | | |
| N | 111 | | | 111 | | |
| Observations | 29,254 |  |  | 3,996 |  |  |
| Marginal R^2^ / Conditional R^2^ | 0.065 / 0.335 | | | 0.005 / 0.144 | | |

*Note*. Bold entries denote statistical significance. *p*-values were computed using Satterwhite correction. CI = Confidence interval; ICC = Intraclass correlation coefficient. τ = Random effects, ρ = correlation between random effects.

**Table S4**

*Model summaries for the selected models of RTs and accuracy during the extinction phase in Experiment 2.*

|  | **RTs** | | | Accuracy | | |
| --- | --- | --- | --- | --- | --- | --- |
| *Predictors* | *Estimates* | *CI* | *p* | *Odds Ratios* | *CI* | *p* |
| (Intercept) | 6.404 | 6.382 – 6.425 | **<0.001** | 13.709 | 12.006 – 15.654 | **<0.001** |
| VMAC | 0.016 | 0.010 – 0.023 | **<0.001** | 0.946 | 0.866 – 1.033 | 0.217 |
| AC | 0.018 | 0.011 – 0.024 | **<0.001** | 0.932 | 0.826 – 1.051 | 0.252 |
| Block | -0.012 | -0.017 to -0.008 | **<0.001** | 1.017 | 0.972 – 1.064 | 0.462 |
| VMAC × Block | -0.005 | -0.010 to -0.000 | **0.044** | 1.136 | 1.039 – 1.241 | **0.005** |
| AC × Block | 0.002 | -0.004 – 0.008 | 0.562 | 0.913 | 0.809 – 1.030 | 0.138 |
| **Random Effects** | | | | | | |
| σ^2^ | 0.0350 | | | 3.290 | | |
| τ_00_ | 0.0132 _Intercept_ | | | 0.433 _Intercept_ | | |
| τ_11_ | 0.0006 _VMAC_ | | |  | | |
|  | 0.0002 _AC_ | | |  | | |
|  | 0.0004 _Block_ | | |  | | |
| ρ_01_ | -0.19 | | |  | | |
|  | 0.32 | | |  | | |
|  | 0.07 | | |  | | |
| ICC | 0.291 | | | 0.12 | | |
| N | 111 | | | 111 | | |
| Observations | 29,330 |  |  | 3,996 |  |  |
| Marginal R^2^ / Conditional R^2^ | 0.006 / 0.288 | | | 0.002 / 0.118 | | |

*Note*. Bold entries denote statistical significance. *p*-values were computed using Satterwhite correction. CI = Confidence interval; ICC = Intraclass correlation coefficient. τ = Random effects, ρ = correlation between random effects.

**Table S5**

*Model summaries for the selected models of RTs and accuracy during the acquisition phase: comparison between experiments.*

|  | | **RTs** | | | **Accuracy** | | |
| --- | --- | --- | --- | --- | --- | --- | --- |
| *Predictors* | | *Estimates* | *CI* | *p* | *Odds Ratios* | *CI* | *p* |
| (Intercept) | 6.553 | | 6.537 – 6.569 | **<0.001** | 16.269 | 14.702 – 18.005 | **<0.001** |
| VMAC | 0.034 | | 0.029 – 0.039 | **<0.001** | 0.933 | 0.860 – 1.013 | 0.100 |
| Block | -0.058 | | -0.062 to -0.054 | **<0.001** | 0.954 | 0.911 – 0.998 | **0.043** |
| Experiment | 0.002 | | -0.029 – 0.034 | 0.883 | 1.426 | 1.167 – 1.743 | **0.001** |
| VMAC × Block | 0.011 | | 0.007 – 0.015 | **<0.001** | 0.962 | 0.897 – 1.033 | 0.285 |
| VMAC × Experiment | 0.013 | | 0.002 – 0.024 | **0.022** | 1.136 | 0.983 – 1.313 | 0.085 |
| Block × Experiment | 0.002 | | -0.007 – 0.010 | 0.683 | 1.061 | 0.975 – 1.154 | 0.171 |
| (VMAC × Block) × Experiment | 0.011 | | 0.004 – 0.019 | **0.003** | 0.944 | 0.823 – 1.084 | 0.417 |
| **Random Effects** | | | | | | | |
| σ^2^ | | 0.043 | | | 3.290 | | |
| τ_00_ | | 0.014 _Intercept_ | | | 0.475 _Intercept_ | | |
| τ_11_ | | 0.001 _VMAC_ | | | 0.020 _VMAC_ | | |
|  | | 0.001 _Block_ | | | 0.0266 _Block_ | | |
| ρ_01_ | | -0.14 | | | 0.04 | | |
|  | | -0.20 | | | -0.12 | | |
| ICC | | 0.26 | | | 0.13 | | |
| N | | 214 | | | 214 | | |
| Observations | | 47,481 | | | 5,136 | | |
| Marginal R^2^ / Conditional R^2^ | | 0.061 / 0.302 | | | 0.010 / 0.142 | | |

*Note*. Bold entries denote statistical significance. *p*-values were computed using Satterwhite correction. CI = Confidence interval; ICC = Intraclass correlation coefficient. τ = Random effects, ρ = correlation between random effects.

**Table S6**

*Model summaries for the selected models of RTs and accuracy during the extinction phase: comparison between experiments.*

|  | | **RTs** | | | **Accuracy** | | |
| --- | --- | --- | --- | --- | --- | --- | --- |
| *Predictors* | | *Estimates* | *CI* | *p* | *Odds Ratios* | *CI* | *p* |
| (Intercept) | 6.412 | | 6.397 – 6.426 | **<0.001** | 16.166 | 14.598 – 17.903 | **<0.001** |
| VMAC | 0.022 | | 0.017 – 0.026 | **<0.001** | 0.983 | 0.904 – 1.069 | 0.691 |
| Block | -0.015 | | -0.018 to -0.012 | **<0.001** | 1.003 | 0.969 – 1.038 | 0.869 |
| Experiment | -0.002 | | -0.031 – 0.027 | 0.907 | 1.461 | 1.193 – 1.788 | **<0.001** |
| VMAC × Block | -0.003 | | -0.006 – 0.000 | 0.069 | 1.040 | 0.971 – 1.115 | 0.260 |
| VMAC × Experiment | 0.011 | | 0.001 – 0.020 | **0.028** | 1.042 | 0.901 – 1.205 | 0.576 |
| Block × Experiment | -0.004 | | -0.010 – 0.002 | 0.157 | 0.990 | 0.924 – 1.061 | 0.778 |
| (VMAC × Block) × Experiment | 0.003 | | -0.003 – 0.010 | 0.337 | 0.839 | 0.731 – 0.963 | **0.013** |
| **Random Effects** | | | | | | | |
| σ^2^ | | 0.035 | | | 3.290 | | |
| τ_00_ | | 0.012 _Intercept_ | | | 0.483 _Intercept_ | | |
| τ_11_ | | 0.001 _VMAC_ | | | 0.022 _VMAC_ | | |
|  | | 0.000 _Block_ | | |  | | |
| ρ_01_ | | -0.12 | | | 0.25 | | |
|  | | 0.06 | | |  | | |
| ICC | | 0.26 | | | 0.13 | | |
| N | | 214 | | | 214 | | |
| Observations | | 47,604 | | | 5,136 | | |
| Marginal R^2^ / Conditional R^2^ | | 0.008 / 0.261 | | | 0.010 / 0.138 | | |

*Note*. Bold entries denote statistical significance. *p*-values were computed using Satterwhite correction. CI = Confidence interval; ICC = Intraclass correlation coefficient. τ = Random effects, ρ = correlation between random effects.

**Table S7**

*Correlations Between VMAC Effect and UPPS-P Scores*

| ***Experiment*** | ***UPPS-P Scores*** | ***VMAC Acquisition*** | |  | ***VMAC Extinction*** | |
| --- | --- | --- | --- | --- | --- | --- |
|  |  | *r* | *p-value* |  | *r* | *p-value* |
| 1 | Negative Urgency | .006 | .952 |  | -.008 | .186 |
|  | Positive Urgency | -.013 | .181 |  | .040 | .232 |
|  | (Lack of) Premeditation | .101 | .309 |  | -.063 | .527 |
|  | (Lack of) Perseverance | .151 | .128 |  | .033 | .740 |
|  | Sensation Seeking | .093 | .352 |  | .032 | .749 |
|  | General Urgency | -.003 | .978 |  | .015 | .882 |
|  | Total Score | .102 | .307 |  | .013 | .896 |
|  | Negative Urgency | -.050 | .606 |  | .097 | .310 |
|  | Positive Urgency | .045 | .641 |  | .238 | **.012** |
|  | (Lack of) Premeditation | .180 | .059 |  | .098 | .307 |
| 2 | (Lack of) Perseverance | .086 | .373 |  | -.043 | .655 |
|  | Sensation Seeking | .061 | .525 |  | -.005 | .956 |
|  | General Urgency | -.006 | .948 |  | .193 | **.041** |
|  | Total Score | .095 | .322 |  | .131 | .172 |

*Note*. General Urgency refers to the average of both Negative and Positive Urgencies (see Billieux et al., 2021; Riley & Smith, 2017). Bold entries show statistical significance (*p < .05).*

**Table S8**

*Comparison of Correlations Between VMAC Effect and NU/PU Scores Across Experiments 1 and 2*

| *Correlations* | *z score* | *p-value* |
| --- | --- | --- |
| VMAC acquisition - NU | 0.40 | 0.69 |
| VMAC acquisition - PU | -0.42 | 0.67 |
| VMAC extinction - NU | -0.76 | 0.45 |
| VMAC extinction - PU | -1.46 | 0.14 |

*Note.* NU: Negative Urgency; PU: Positive Urgency.

## Power analysis

We performed a simulation-based power analysis using their results as a reference. We assumed the same model structure and effect sizes observed in Garre-Frutos et al. (2024) and then we tested the significance of the VMAC effect and its interaction with Blocks of trials in 1000 simulations with varying sample sizes (from 20 to 120). Figure S1 shows the proportion of significant results as a function of the statistical test and the number of participants. The power analysis shows that at least 100 participants would be necessary to achieve at least 90% power to detect both the VMAC effect and its interaction with the block of trials.


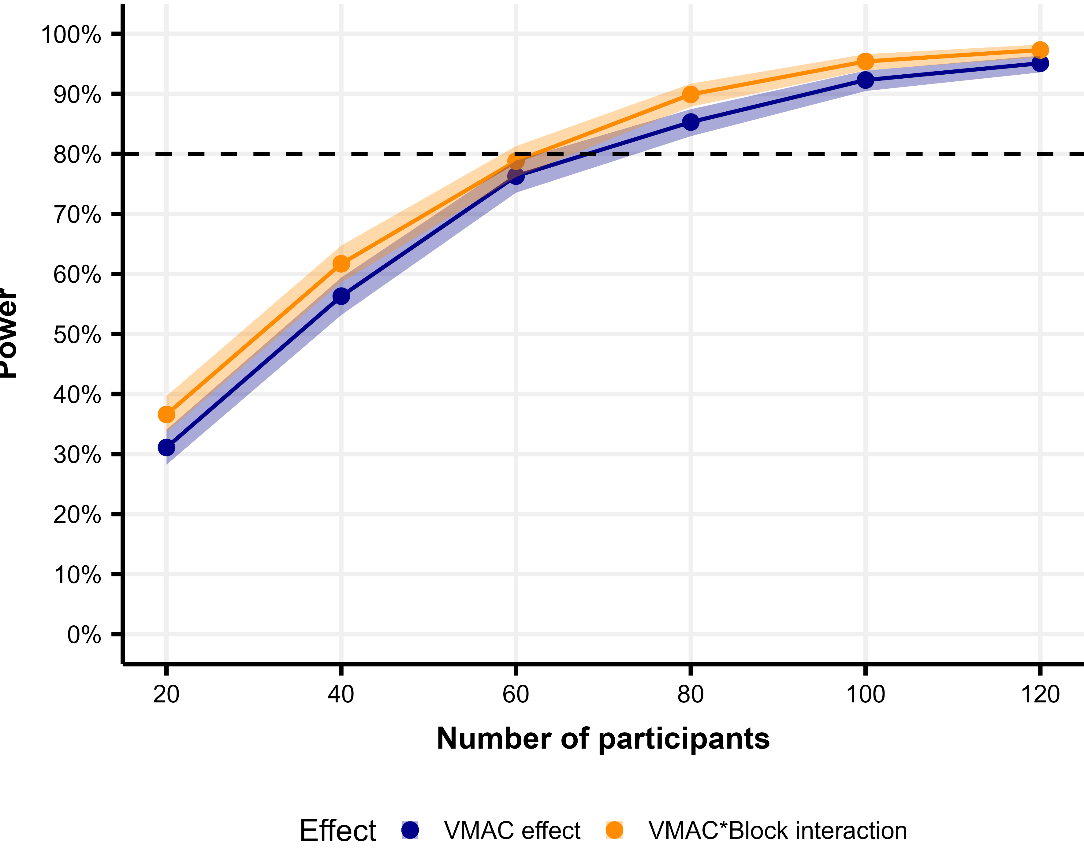


*Figure S1*. Power curve for the VMAC effect and its interaction with Block predictor. Dots indicated the observed proportion of significant results as a function of the effect and the number of participants employed in the simulation. Shaded segments represent the 95% CI for a binomial proportion.

We did not perform a power analysis for the correlational analysis reported in the main text. Nevertheless, we performed a sensitivity analysis. With the sample size of Experiment 1, we would have 80% statistical power to detect a correlation of .268, and in Experiment 2 we would have power to detect a correlation of at least 0.259.
